# Identification of two novel large deletions in *FBN1* gene by next-generation sequencing and multiplex ligation-dependent probe amplification

**DOI:** 10.1186/s12920-024-01822-w

**Published:** 2024-02-05

**Authors:** Xinxin Lu, Ren Wang, Mingjie Li, Biao Zhang, Huiying Rao, Xiaoli Huang, Xijun Chen, Yan’an Wu

**Affiliations:** 1 Center of Clinical Laboratory, Zhongshan Hospital of Xiamen University, School of Medicine, Xiamen University, Xiamen, 361004 China; 2 Department of Cardiovascular Surgery, Fujian Provincial Hospital, Fuzhou, 350001 Fujian China; 3 Department of Clinical Laboratory, Fujian Provincial Hospital, Fuzhou, 350001 Fujian China; 4 Department of Ophthalmology, Fujian Provincial Hospital, Fuzhou, 350001 Fujian China

**Keywords:** NGS, *FBN1*, Large genomic deletions, Marfan syndrome, MLPA

## Abstract

**Background:**

Mutations in *fibrillin-1* (*FBN1*) are known to be associated with Marfan syndrome (MFS), an autosomal dominant connective tissue disorder. Most *FBN1* mutations are missense or nonsense mutations. Traditional molecular genetic testing for the *FBN1* gene, like Sanger sequencing, may miss disease-causing mutations in the gene’s regulatory regions or non-coding sequences, as well as partial or complete gene deletions and duplications.

**Methods:**

Next-generation sequencing, multiplex ligation-dependent probe amplification and gap PCR were conducted on two MFS patients to screen for disease-causing mutations.

**Results:**

We identified two large deletions in *FBN1* from two MFS patients. One patient had a 0.23 Mb deletion (NC_000015.9:g.48550506_48779360del) including 5’UTR-exon6 of *FBN1*. The other patient harbored a 1416 bp deletion (NC_000015.9:g.48410869_48412284del) affecting the last exon, exon 66, of the *FBN1* gene.

**Conclusion:**

Our results expanded the number of large *FBN1* deletions and highlighted the importance of screening for large deletions in *FBN1* in clinical genetic testing, especially for those with the classic MFS phenotype.

## Background

Marfan syndrome (MFS; MIM# 154700) is a heritable autosomal dominant disorder of connective tissues that mainly involves the ocular, skeletal, and cardiovascular systems, with an estimated prevalence of 1:5000 [1]. MFS is caused by mutations in the *FBN1* gene, which is located on chromosome 15q21.1 and encodes a 320-kDa extracellular matrix glycoprotein fibrillin-1, a major component of microfibrils. *FBN1* is a large gene containing 66 exons. Patients with MFS present with a diverse range of clinical manifestations, ranging from isolated features to severe multiorgan involvement. This variability in symptoms can be observed even among family members who share the same *FBN1* mutation [2]. Cardiovascular disorders in MFS such as artery dissection can be life-threatening, even in young adults. The diagnosis of MFS is based on the revised Ghent criteria [3]. Certain diseases, like Loeys-Dietz syndrome and the vascular form of Ehlers-Danlos syndrome, may present similar symptoms and phenotypes as MFS. When specific clinical manifestations unique to Marfan syndrome, such as lens dislocation, are not present, the only reliable method to distinguish between these disorders is through genetic testing [3]. Because disease management and treatment guidelines vary for MFS and its related syndromes, the correct diagnosis is of clinical significance.

To date, over 3000 mutations scattered across the *FBN1* gene have been reported by the Human Gene Mutation Database (HGMD, https://www.hgmd.cf.ac.uk/), with missense mutations being predominant [4, 5]. Nonetheless, overlooking large deletions and duplications would be a critical omission. Currently, next-generation sequencing (NGS) is considered a powerful technology for genetic screening in clinical disease. Multiplex ligation-dependent probe amplification (MLPA) is a commonly used technique for rapidly and conveniently screening large deletions and duplications. It is widely utilized due to its ease of use and efficiency. Hence, an increasing number of large genomic rearrangements in *FBN1* have been discovered (summarized in Table 1) [5–14].

**Table 1:** Summary of MFS cases with large deletions in the FBN1 gene

Variation	Affected domains	Patient age (y)	Phenotype in papers	Reference PMID (year)
Deletion (FBN1 exon affected)				
Single-exon deletion				
FBN1:g.46,701,985_46,728,871(Ex1)	–	25	Classic MFS	17,492,313(2002)
FBN1:Ex1	–	U	Classic MFS	24,501,682(2013)
FBN1:Ex1	–	U	Classic MFS	24,793,577(2014)
FBN1:Ex2	–	52	Classic MFS	11,700,157(2001)
FBN1:Ex3	1st EGF-like	U	MFS	21,907,952(2011)
FBN1:Ex6	3rd EGF-like	49	Potential MFS	28,842,177(2017)
FBN1:c.3603_3668del(Ex29)	18th cbEGF-like	After birth	Neonatal MFS	10,441,700(1999)
FBN1:Ex30	19-20th cbEGF-like	< 1	Suspected Beals-Hecht syndrome	25,944,730(2015)
FBN1:Ex32	21-22th cbEGF-like	1	Neonatal MFS	18,412,115(2008)
FBN1:Ex36	25-26th cbEGF-like	U	Classic MFS	19,839,986(2009)
FBN1:g.48,749,026_48,753,819del(Ex43)	7th TB,29th cbEGF-like	24	Classic MFS	30,286,810(2018)
FBN1:g.48,734,801_48,730,690(Ex50)	35th cbEGF-like	14	MFS	30,286,810(2018)
FBN1:Ex66	COOH unique region	47	Classic MFS	Current report
Multiple exon deletion				
FBN1:Ex1–5	1-3rd EGF-like	27	Classic MFS	21,936,929(2011)
FBN1:Ex1–6	1-3rd EGF-like	22	Classic MFS	Current report
FBN1:g.46,580,456_46,883,035(Ex1–16)	1-3rd EGF-like,1st TB,4-10th cbEGF-like	40	Classic MFS	17,492,313(2002)
FBN1:Ex1–36	1-3rd EGF-like,1–5th TB,4-26th cbEGF-like	15	Classic MFS	28,842,177(2017)
FBN1:g.48,890,962_48,922,918(Ex2–4)	1-2nd EGF-like	32	Classic MFS	29,850,152(2018)
FBN1:Ex6–65	3rd EGF-like,4-47th cbEGF-like,1-9th TB	U	Classic MFS	24,793,577(2014)
FBN1:Ex13–15	7-10th cbEGF-like	U	MFS	33,436,942(2021)
FBN1:Ex13–49	7-34th cbEGF-like,3-7th TB	5	MFS	18,412,115(2008)
FBN1:Ex18–22	3-4th TB,11-13th cbEGF-like	U	MFS	31,730,815(2020)
FBN1:Ex23–25	4-5th TB,14-15th cbEGF-like	U	MFS	34,325,513(2021)
FBN1:Ex24–26	14-16th cbEGF-like	After birth	Neonatal MFS	20,455,198(2010)
FBN1:Ex33–38	21-26th cbEGF-like,6th TB	1	Neonatal MFS	24,199,744(2014)
FBN1:Ex34–43	23-29th cbEGF-like,6-7th TB	22	Classic MFS	19,863,550(2010)
FBN1:Ex37–65	26-47th cbEGF-like,3-9th TB	U	Classic MFS	24,793,577(2014)
FBN1:Ex42–43	7th TB,29th cbEGF-like	> 46	Classic MFS	11,710,961(2001)
FBN1:Ex44–46	29-31th cbEGF-like	> 6	Childhood onset MFS	11,710,961(2001)
FBN1:Ex44–66	23-47th cbEGF-like,8-9th TB	37	Classic MFS	30,286,810(2018)
FBN1:Ex46–47	31-32th cbEGF-like	U	Juvenile onset MFS	36,945,115(2023)
FBN1:Ex48–53	33-37th cbEGF-like,8 TB	15	Neonatal MFS	28,842,177(2017)
FBN1:Ex49–50	34-35th cbEGF-like	3	Neonatal MFS	28,842,177(2017)
FBN1:Ex50–63	35-46th cbEGF-like,8-9th TB	65	MFS	19,659,760(2009)
FBN1:Ex58–63	41-46th cbEGF-like	17	Juvenile onset classic MFS	17,189,636(2007)
FBN1:Ex61–63	43-46th cbEGF-like	48	Classic MFS	1,631,074(1992)
FBN1:Ex1–66	Full gene	16	Incomplete MFS	20,478,419(2010)
FBN1:Ex1–66	Full gene	42	Classic MFS	21,936,929(2011)
FBN1:Ex1–66	Full gene	15	Classic MFS	21,936,929(2011)
FBN1:Ex1–66	Full gene	12	Classic MFS	21,936,929(2011)
FBN1:Ex1–66	Full gene	41	MFS	21,063,442(2011)
FBN1:Ex1–66	Full gene	39	MFS	21,063,442(2011)
FBN1:Ex1–66	Full gene	16	MFS	21,063,442(2011)
FBN1:Ex1–66	Full gene	13	MFS	21,063,442(2011)
FBN1:Ex1–66	Full gene	27	MFS	21,063,442(2011)
FBN1:Ex1–66	Full gene	21	MFS	21,063,442(2011)
FBN1:Ex1–66	Full gene	34	MFS	21,063,442(2011)
FBN1:Ex1–66	Full gene	5	Potential MFS	21,063,442(2011)
FBN1:Ex1–66	Full gene	13	Potential MFS	21,063,442(2011)
FBN1:Ex1–66	Full gene	8	Potential MFS	21,063,442(2011)
FBN1:Ex1–66	Full gene	13	Classic MFS	22,260,333(2012)
FBN1:g.48,931,968_51,102,375(Ex1–66)	Full gene	14	MFS	27,615,407(2016)

In this study, we detected two large deletions in *FBN1* using NGS in patients with MFS. Validation of these deletions was conducted using multiplex ligation-dependent probe amplification (MLPA), while the deletion breakpoints were characterized through gap PCR or whole-genome sequencing (WGS). Our results expand the number of large *FBN1* deletions and highlighted the necessity of screening for large deletions in *FBN1* in clinical genetic testing, especially in individuals displaying classic MFS phenotype.

## Methods

### Editorial policies and ethical considerations

This study was approved by the Institutional Review Board of Fujian Provincial Hospital (K2015–02-022) and performed in accordance with the Helsinki declaration and its later amendments or comparable ethical standards. Informed consent was obtained from all the participants**.**

### Participants

Patients with MFS and their family members referred for a genetic test from the Department of Cardiovascular Surgery in Fujian Provincial Hospital (Fuzhou, Fujian Province, China) were recruited.

### Targeted next-generation sequencing

Total genomic DNA was extracted using a blood DNA extraction kit (QIAamp, Germany) according to the manufacturer’s protocol. Potential mutations were analyzed using a customized capture array (Roche NimbleGen Inc., Madison, WI, USA) and the Illumina HiSeq 2500 platform by BGI (Shenzhen, China), as previously reported [15]. The target region for this study encompassed all exons and an additional 20 base pairs of the adjacent intronic regions of three genes: *FBN1*, *TGFBR1*, and *TGFBR2*. DNA template libraries were prepared according to the manufacturer’s recommendation. Equal molar ratios of 10 indexed samples were pooled and loaded onto each lane of the flow cells for sequencing with 100-cycle single-end reads. Raw data in the base call files (.bcl format) were converted to qseq files before demultiplexing with CAVAv1.7 software (Illumina Inc., San Diego, CA, USA). Demultiplexed data were further processed by NextGENe software for alignment (SoftGenetics, State College, PA, USA). The average depth of coverage of the NGS analysis was 500–1000×. All exons were covered to a sufficient depth [16]. Coverage-based depth analysis using NGS data has been previously reported [17]. Exon deletion was identified via CNV detection using a statistical algorithm in the workflow, as reported in 2014 [18, 19].

### Multiplex ligation-dependent probe amplification

To confirm large deletions or duplications in *FBN1*, MLPA assays were performed using the commercially available SALSA MLPA kits P065-B1 and P066-B1 (MRC-Holland, Amsterdam, The Netherlands), which contain probes for all exons of *FBN1*. According to the manufacturer’s instructions, a total of 100–200 ng of genomic DNA obtained from each patient was used for hybridization, and amplification products from each MLPA assay were separated using capillary electrophoresis with an ABI 3130 Genetic Analyzer (Life Technologies, Carlsbad, CA, USA). The results were analyzed using Coffalyser software.

### Whole-genome sequencing

Genomic DNA extracted from blood was used to perform WGS for Patient 1 via the commercial provider Macrogen (South Korea) using Illumina HiSeqX technology. Sequencing was performed to an average sequence depth of 28.5×. The resulting sequence files were aligned to hg38 using Isaac Aligner [20]. Subsequently, WGS data were analyzed for CNVs using the software Nexus Copy Number (BioDiscovery, El Segundo, CA, USA).

### Identification and validation of breakpoints

Based on the results of WGS and MLPA, deletions were confirmed via identification of respective breakpoints using gap PCR, followed by Sanger sequencing (ABI 3130, Life Technologies) for Patients 1 and 2. This same procedure was also carried out for the son of Patient 2.

## Results

### Clinical findings

The clinical manifestations of the patients and family history are summarized in Table 2. Patient 1 was 184 cm in height and 70 kg in weight. He presented with classic MFS involving a wide range of thoracic and abdominal aortic dissections, joint hypermobility, positive thumb and wrist signs, bilateral ectopia lentis, dural ectasia and marked diffuse striae over the lower back and hips. His arm span-to-height ratio was 1.01. At the age of 23, the echocardiography examination revealed dilatation of the ascending aorta with a diameter of 4.95 cm (Z score > 2.0) at the sinus of Valsalva, severe mitral valve prolapse with valve regurgitation and global cardiac enlargement. Computerized tomography revealed right pulmonary bullae. Patient 1 was diagnosed with Marfan syndrome based on the clinical features of aortic dilatation and bilateral ectopia lentis. Although the proband’s parents had no features of MFS, they were not available for gene testing. Patient 2 was 172 cm in height and 60 kg in weight. She exhibited ascending aorta dissection, joint hypermobility, positive thumb and wrist signs and dural ectasia. At the age of 46, the echocardiography examination showed an enlargement of the ascending aorta with a diameter of 4.9 cm (Z score > 2.0) at the sinus of Valsalva, along with aortic valve regurgitation. After complete mydriasis, the patient underwent a slit-lamp examination, during which no dislocation of the lens was detected. However, she refused to undergo ultrasound biomicroscopy, the possibility of slight lens dislocation cannot be entirely eliminated. The proband’s 20-year-old son was 192 cm in height and 86 kg in weight. His span-to-height ratio was 0.98. He exhibited positive thumb and wrist signs, a high palate and diffuse striae. A vision test revealed that he was narsighted with 4 diopters. He was unavailable for other examinations. The patient’s parents have both passed away, with the mother’s death being sudden. Additionally, the patient’s older sister died from aortic dissection.

**Table 2:** Clinical profiles of the patients

Organ systems	Criteria	P1	P2
Cardiovascular	Enlarged aortic diameter or aortic dissection	Y	Y
	Mitral valve prolapsed with valve regurgitation	Y	N
Skeletal	Pectus carinatum	N	N
	Wrist and thumb signs	Y	Y
	Scoliosis of > 20°	N	N
	Reduced upper-segment to lower-segment ratio or arm span-to-height ratio > 1.05	N	N
	High palate	NA	N
	Reduced extension at the elbows	N	N
	Joint hypermobility	Y	Y
Ocular	Ectopia lentis	Y	N
	High myopia	Y	N
Skin	Striae	Y	N
Pulmonary		Y	NA
Dural		Y	Y
Height (cm)		184	172
Weight (kg)		70	60
Age at presentation (years)		23	46
Sex		Male	Female
Family history		N	N

*P* patient, *N* absence of criterion, *NA* not available, *P* patient, *Y* presence of criterion

Patients 1 and 2 received a total aortic arch replacement with the stented elephant trunk technique to treat artery dissections.

### Targeted next-generation sequencing

Two large heterozygous deletions, EX1_6 DEL and EX66 DEL, were found in Patient 1 and Patient 2, respectively.

### Multiplex ligation-dependent probe amplification

**Fig. 1:**
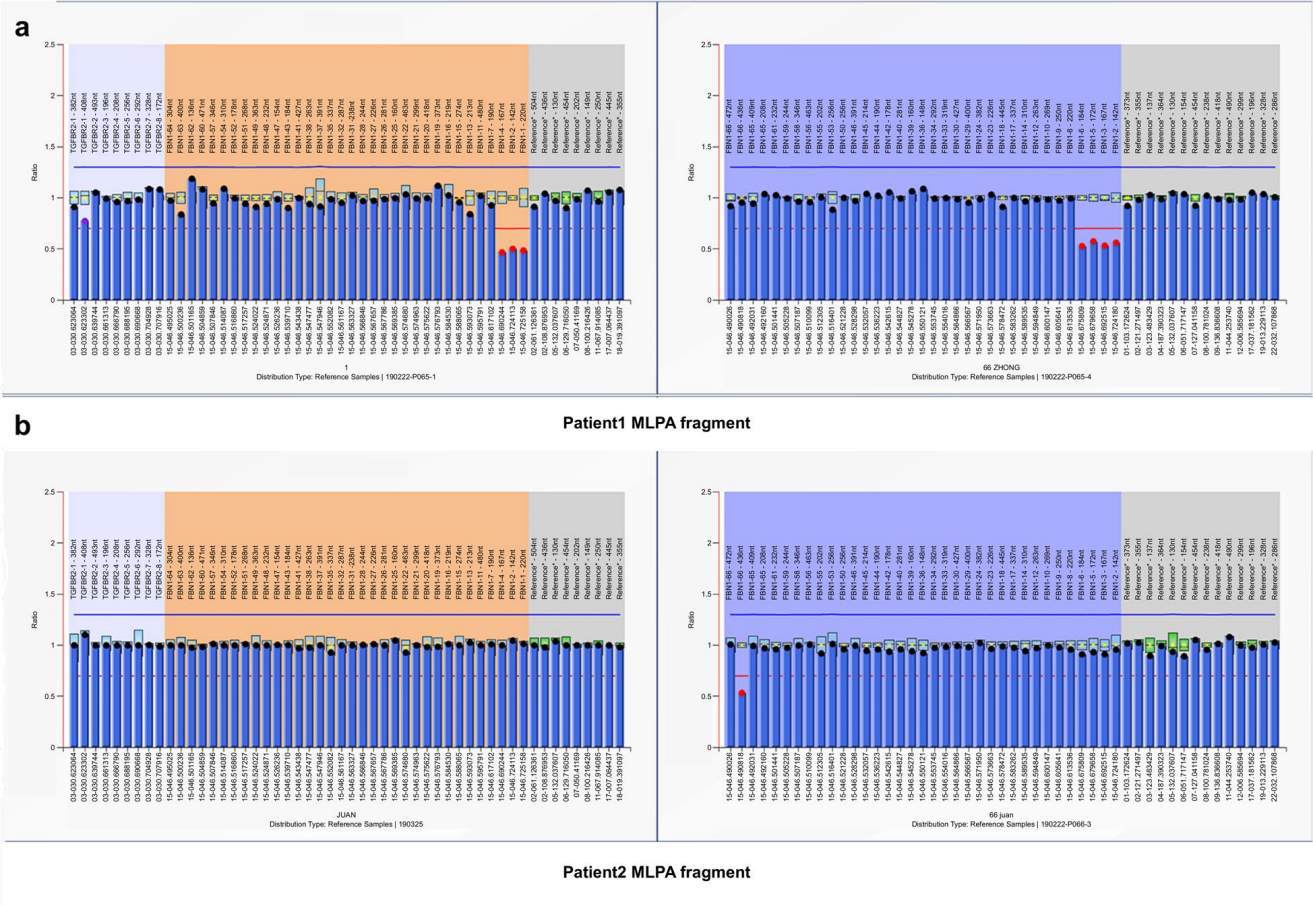
Results of semiquantitative MLPA analyses for two patients. Normalized relative peak areas measured with P065 and P066 kits-B1. **a** Reduced relative peak areas of *FBN1* exons 1–6 for Patient 1. **b** Reduced relative peak areas of *FBN1* exon 66 for Patient 2

For Patient 1, MLPA showed reduced relative peak areas of fragments corresponding to exons 1–6 (Fig. 1a), suggesting a heterozygous deletion of these exons.

For Patient 2, MLPA revealed reduced a relative peak area for the 436 nucleotide (nt) probe of *FBN1* exon 66, suggesting a heterozygous deletion of exon 66, while the peak area for the 472 nt probe of *FBN1* exon 66 remained normal (Fig. 1b).

### Whole-genome sequencing

**Fig. 2:**
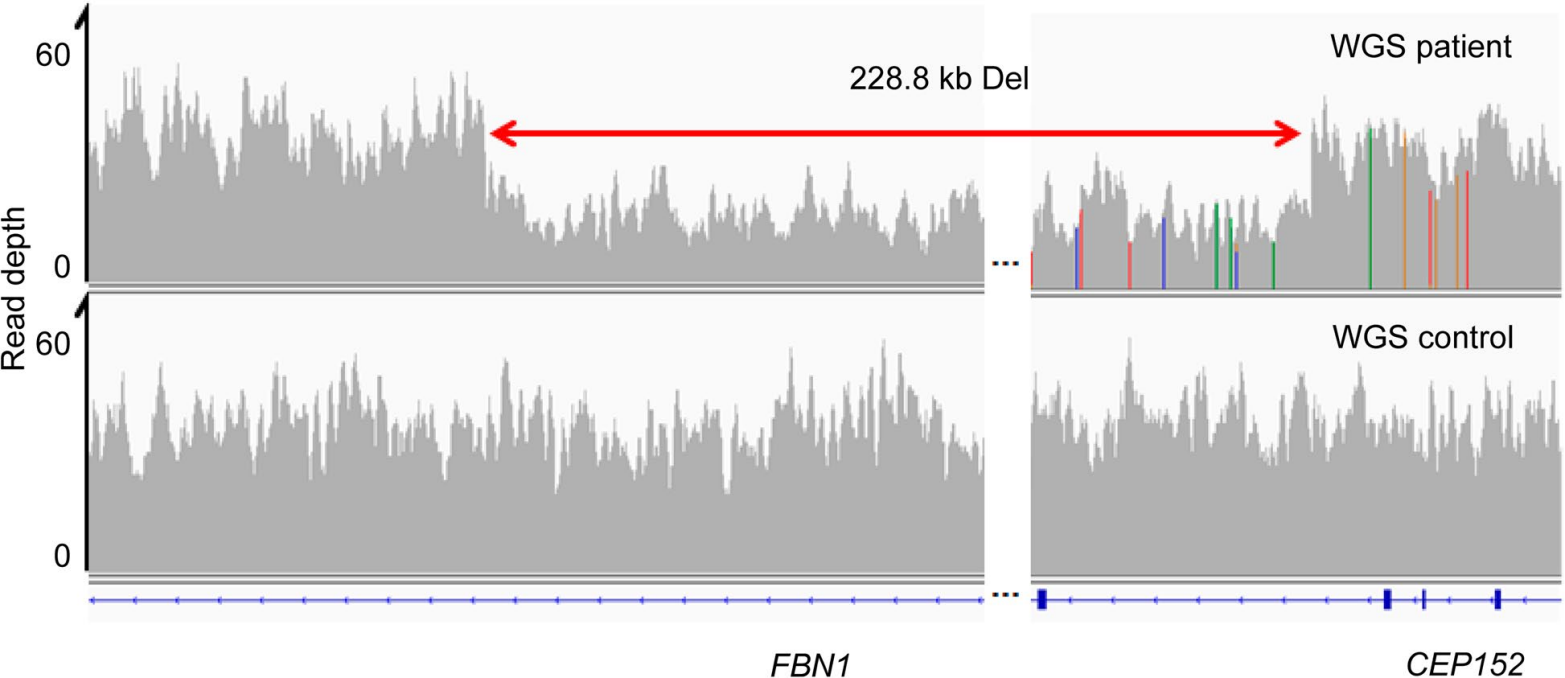
CNV detection by NGS. Read depth (coverage tracks) of 60× PE150 PCR-free WGS data for Patient 1 and a control for the deleted and flanking genomic regions displayed in IGV. CNV, copy number variation; NGS, next-generation sequencing; WGS, whole-genome sequencing; IGV, Integrative Genomics Viewer [http://www.broadinstitute.org/igv/, accessed Dec 2016]

A 0.23 Mb deletion (NC_000015.9:g.48550506_48779360del) including 5’UTR-exon6 of *FBN1* was discovered (Fig. 2). The mutation has not been reported in the Human Gene Mutation Database (HGMD, https://www.hgmd.cf.ac.uk/) or ClinVar (https://www.ncbi.nlm.nih.gov/clinvar/). Based on the ACMG guidelines for genetic variant classification [21], this deletion meets one very strong pathogenic evidence (the deletion may cause nonsense-mediated mRNA decay), one moderate pathogenic evidence (variant not reported on Decipher (https://www.deciphergenomics.org/) and IGV (https://igv.org)) and one supporting evidence (the patient’s phenotype is consistent with the phenotype caused by *FBN1* in MFS), namely PVS1 + PM2_Supporting+PP4. Therefore, it can be classified as a pathogenic(P) variant.

### Identification and validation of breakpoints

**Fig. 3:**
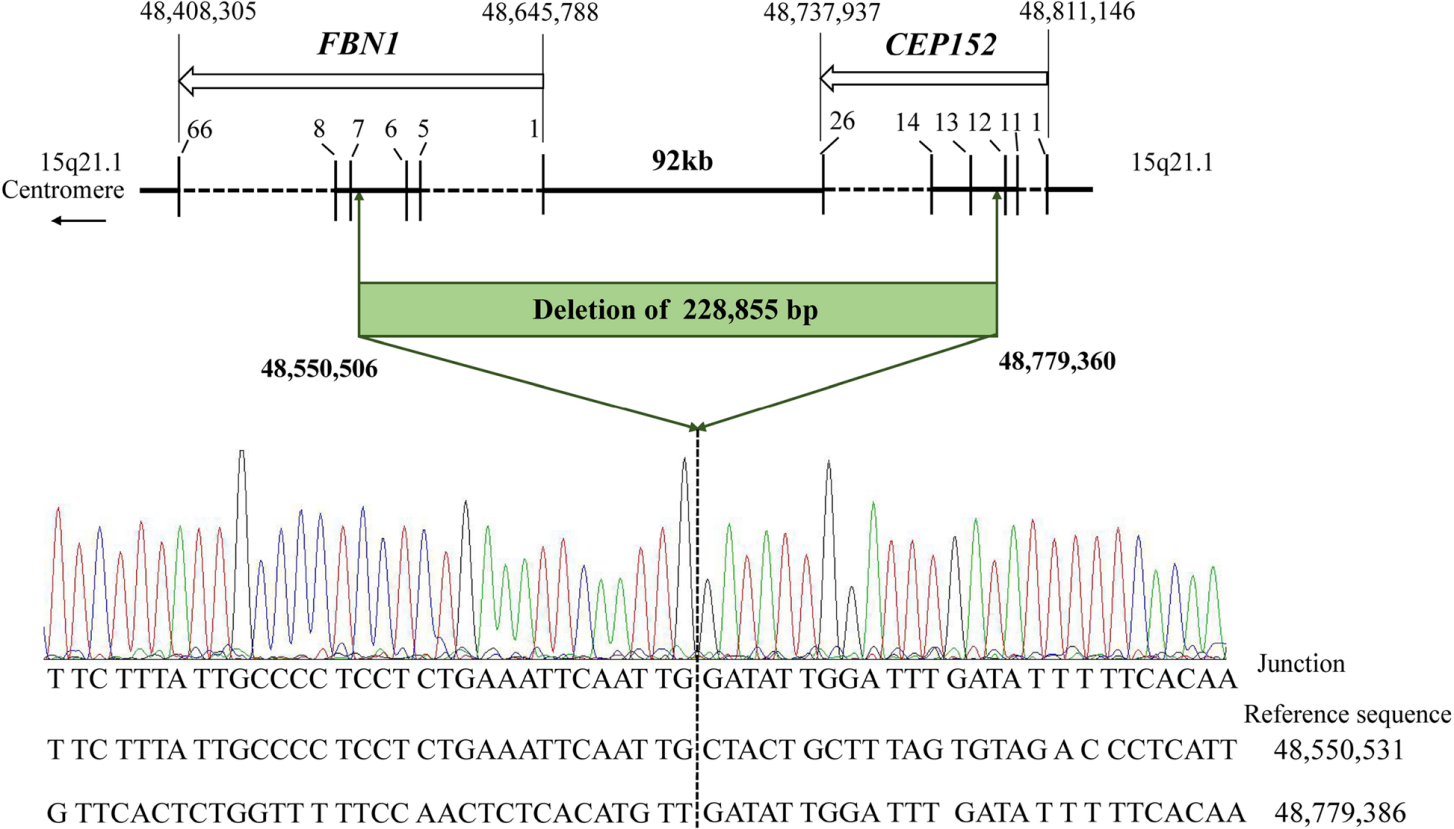
Identification of the breakpoints in Patient 1. Schematic representation of the genome in the deletion region as well as an overview of the results of Sanger sequencing analyses. The open arrow below the gene name indicates the direction of transcription. Exons are specified by bars and labeled with the corresponding number. The dotted line marks the position of the breakpoints. The left side of the dotted line represents the sequence of *FBN1* intron 6; the other side of the dotted line represents the sequence of *CEP152* intron 12. Nucleotide positions are described in relation to the human genome reference sequence GRCh38.WGS data are displayed in the National Center for Biotechnology Information (NCBI https://www.ncbi.nlm.nih.gov/bioproject)

The deletion in Patient 1 was reconfirmed using gap PCR spanning deletion breakpoints, followed by Sanger sequencing (Fig. 3).

**Fig. 4:**
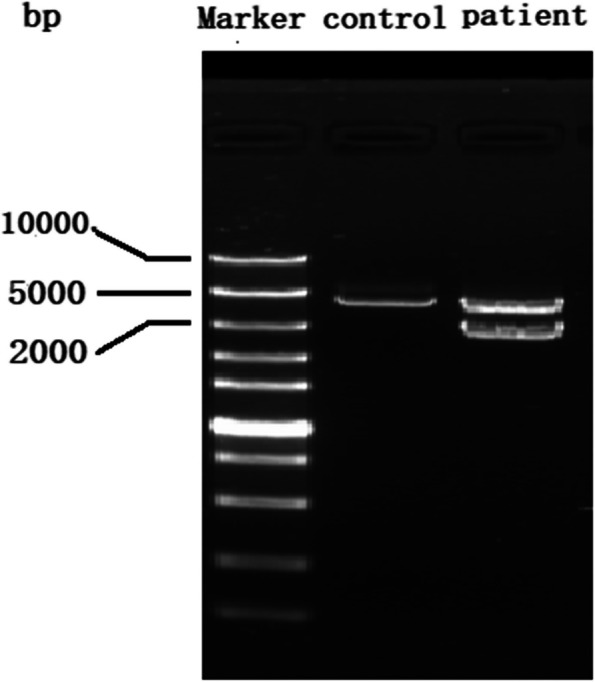
Gap PCR products for Patient 2 and the control

**Fig. 5:**
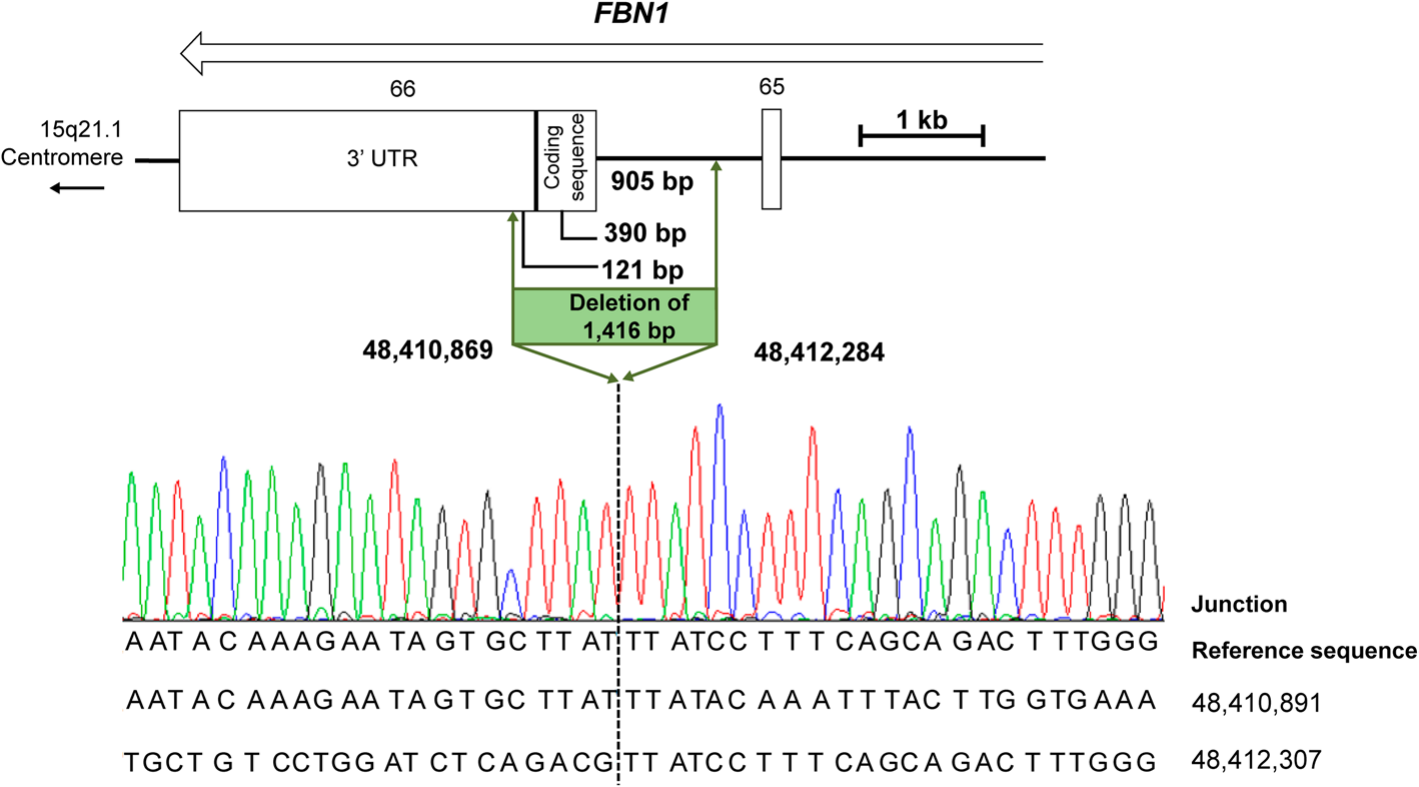
Identification of the breakpoints in Patient 2. Schematic representation of the genome in the deletion region as well as an overview of the results of Sanger sequencing analyses. The open arrow below the gene name indicates the direction of transcription. Exons are represented by rectangles and labeled with the corresponding number. The dotted line marks the position of the breakpoint. Nucleotide positions are described in relation to the human genome reference sequence GRCh38

Similarly, the deletion in Patient 2 was confirmed through gap PCR. Agarose gel analyses showed the presence of a shorter band, in addition to the normal band, among the PCR products (Fig. 4). Further Sanger sequencing of the shorter band identified a deletion of 1416 bp (NC_000015.9:g.48410869_48412284del) in *FBN1* exon 66 (Fig. 5). Sanger sequencing confirmed that Patient 2’s son carried the same mutation inherited from his mother. The deletion has not been reported in the Human Gene Mutation Database (HGMD, https://www.hgmd.cf.ac.uk/) or ClinVar (https://www.ncbi.nlm.nih.gov/clinvar/). Based on the ACMG guidelines for genetic variant classification, this deletion meets one very strong pathogenic evidence and one moderate pathogenic evidence (variant not reported on Decipher and IGV), namely PVS1 + PM2_Supporting. Therefore, it can be classified as a likely pathogenic (LP) variant.

## Discussion

In this study, we initially applied targeted NGS to screen for mutations in MFS patients, followed by subsequent validation using MLPA. Breakpoints were defined via WGS and Sanger sequencing. As a result, two novel large deletions within *FBN1*, one spanning exons 1–6 and the other in exon 66, were identified in two unrelated patients. Our results emphasize the significance of screening for large genomic mutations in MFS and further extend the mutation spectrum of *FBN1* associated with MFS.

Despite numerous attempts using gap PCR, we failed to identify the breakpoints in Patient 1. However, the breakpoint was ultimately detected using WGS. In a study by Benke et al., they reported a patient with MFS who had a 32-kb *FBN1* deletion that was identified using WGS [11]. In our work, WGS confirmed that Patient 1 harbored a deletion of 0.23 Mb, spanning from intron 12 of *CEP152,* an upstream gene neighboring *FBN1*, to intron 6 of *FBN1*. *CEP152* consists of 26 exons and encodes a 152 kDa centrosomal protein that may have played an important role in the evolution of human brain size [22]. Since disease-causing variants in this gene cause autosomal recessive disorders (OMIM# 614852 and OMIM# 613823), Patient 1 do not exhibit phenotypes other than MFS. Previous studies have reported deletions beyond exon 1 of *FBN1* [23–25]. It is worth noting that the mutation identified in patient 1 closely resembles the mutation described in one of these studies [23]. Both mutations involve deletions of the *CEP152* and *FBN1* genes, although the specific mutation breakpoints differ.

In Patient 1, deletion of exons 1–6 in *FBN1* eliminates the ATG initiation codon and a putative promoter sequence in its upstream region. Therefore, this deletion possibly results in a defective *FBN1* transcript and leads to a single functional *FBN1* allele. These findings suggest that *FBN1* haploinsufficiency may play a role in the development of MFS in this patient. Patient 1 exhibited a classic MFS phenotype characterized by multiple systemic deformities. A few studies have reported some heterozygous deletions of *FBN1*. In a study from 2011, a patient was identified with a deletion spanning from exon 1 to exon 5 [24]. Another study in 2007 reported two patients carrying deletions affecting exons 1 and 1–16 [23]. Furthermore, a patient described in 2017 harbored a deletion involving exons 1–36 [14]. These patients had only one functional *FBN1* allele, and each presented classical MFS phenotypes.

The investigation into the correlation between the phenotype and genotype of Marfan syndrome has been an ongoing endeavor. Gergely et al. found that abolishment of regulatory elements by a deletion (such as lack of a transcription-binding site for STAT3) may lead to more severe manifestations and seems to play a role in the development of the cardiovascular phenotype in this monogenic disorder [8]. Their data analyses on previously published CNVs demonstrated the presence of a potential transcription-binding site for STAT3 in five of 25 patients [8]. One of these patients had a deletion that affected both exon 1 and exon 2, as well as the promoter region, similar to Patient 1 in the current study. Therefore, deletion affecting the STAT3-binding site in Patient 1 may have resulted in severe cardiovascular symptoms.

Patient 1 presented with dural ectasia, a feature of little concern in MFS. Dural ectasia is a widening of the dural sac with bony erosions of the vertebral bodies [26]. There is no consistent relationship of dural ectasia with any specific type of *FBN1* mutation [27].

Patient 2 harbored a 1416 bp deletion (NC_000015.9:g.48410869_48412284del) of *FBN1* exon 66. This deletion, which was confirmed using gap PCR and Sanger sequencing, involved 905 bp of intron 65 and 511 bp of exon 66 (390 bp of coding sequence and 121 bp of the 3’UTR). MLPA findings revealed a reduced relative peak area for the 436 nt probe of *FBN1* exon 66, while the peak area for the 472 nt probe of *FBN1* exon 66 remained normal. This observation suggests that exon 66 is partially deleted, resulting in the probe’s inability to bind and interact with the target sequence. As a consequence, amplification cannot occur, leading to a positive result. The findings from MLPA were consistent with those from gap PCR. Deletion of 390 bp in the coding region may lead to shortening of the *FBN1* molecule; deletion in the 3’UTR may have an impact on patient phenotype. A 3’UTR mutation in *FBN1* was identified in patients with MFS, the molecular mechanism of which suggests the involvement of endoplasmic reticulum stress responses in the formation of aortic aneurysms [28]. The 3’UTR is a region of the mRNA molecule that does not code for proteins but plays a crucial role in gene regulation and mRNA stability. Mutations in this region may affect gene expression and lead to disease phenotypes. It is still unclear whether the truncated mRNA formed by the partial deletion of exon 66 in Patient 2 will trigger the nonsense-mediated mRNA decay. Whether Patient 2 with MFS is caused by dominant negative effects also requires further investigation.

Ultrasound biomicroscopy is an advanced diagnostic technique developed in recent years. It is considered the gold standard for diagnosing lens dislocation as it can directly detect ruptures in the lens zonules [29]. In some clinical cases, it can be challenging to identify mild lens subluxation, even under slit lamp examination where iridodonesis may not be apparent. In such situations, ultrasound biomicroscopy examination can be employed to assess the condition of the zonules in a 360° range, allowing for the detection of minor and subtle incomplete lens dislocations. Patient 2 refused to undergo ultrasound biomicroscopy, the possibility of slight lens dislocation cannot be entirely eliminated.

The MLPA method is commonly employed to detect abnormal CNVs in specific genomic DNA or RNA sequences. However, it does have certain technical limitations. First, it is primarily suited for detecting gene deletions or duplications and is not designed to detect point mutations directly. Second, it cannot be used for single-cell detection. Additionally, there is a risk of false positive results if a point mutation is located at the end of the probe. As a result, the MLPA method is limited to the detection of specific target genes.

## Conclusions

Our report expanded the number of large *FBN1* deletions and highlighted the importance of screening for large deletions in *FBN1* in clinical genetic testing, especially for those with the classic Marfan phenotype.

## Data Availability

The datasets generated and analyzed during the current study are available in the NCBI Sequence Read Archive repository, https://www.ncbi.nlm.nih.gov/bioproject/PRJNA719684.

## Abbreviations

FBN1: Fibrillin-1
NGS: Next-generation sequencing
MLPA: Multiplex ligation-dependent probe amplification
MFS: Marfan syndrome
PCR: Polymerase chain reaction
WGS: Whole genome sequencing
CNV: Copy number variation
